# Successful endovascular treatment with a stent graft for chronic deep vein thrombosis with multiple arteriovenous fistulas: a case report

**DOI:** 10.1186/s13256-022-03480-x

**Published:** 2022-07-02

**Authors:** Tamon Kato, Megumi Fuke, Fumio Nagai, Hidetomo Nomi, Yusuke Kanzaki, Hisanori Yui, Shusaku Maruyama, Ayumu Nagae, Takahiro Sakai, Tatsuya Saigusa, Soichiro Ebisawa, Ayako Okada, Hirohiko Motoki, Koichiro Kuwahara

**Affiliations:** 1 Department of Cardiovascular Medicine, Shinshu University School of Medicine, 3-1-1 Asahi, Matsumoto, Nagano 390-8621 Japan; 2 Division of Cardiovascular Surgery, Department of Surgery, Shinshu University School of Medicine, Matsumoto, Japan; 3 Department of Plastic and Reconstructive Surgery, Shinshu University School of Medicine, Matsumoto, Japan

**Keywords:** Chronic deep venous thrombosis, Arteriovenous fistula, Endovascular treatment, Stent graft

## Abstract

**Background:**

Deep vein thrombosis with arteriovenous fistulas is rare, with few therapeutic options available for chronic-phase deep vein thrombosis. Moreover, the effectiveness of endovascular treatment for chronic-phase deep vein thrombosis with arteriovenous fistulas has not been established. We describe herein a case of successful endovascular treatment for chronic deep vein thrombosis with multiple arteriovenous fistulas.

**Case presentation:**

We describe the case of a 72-year-old Asian woman who had begun experiencing left leg swelling and intermittent claudication 2 years prior. Enhanced computed tomography revealed left common iliac vein occlusion with vein-to-vein collateral formation and several arteriovenous fistulas. Angiography and ultrasound showed the arteriovenous fistulas to run from the common and internal iliac arteries to the external iliac and superficial femoral veins. We opted against surgical repair for the arteriovenous fistulas due to their complex nature and complicated morphology. Since her condition was progressive, endovascular treatment with a stent graft was performed for the deep vein thrombosis, after which her symptoms gradually improved. Four months following the procedure, enhanced computed tomography confirmed remarkable reduction of the vein-to-vein collaterals and arteriovenous fistulas.

**Conclusions:**

In the present case, enhanced computed tomography with a stent graft was effective in improving symptoms. This strategy may therefore be a treatment option for intractable chronic deep vein thrombosis with arteriovenous fistulas.

## Introduction

Post-thrombotic syndrome (PTS) reportedly develops in 20–50% of deep vein thrombosis (DVT) cases [1], with many patients inconvenienced by chronic DVT symptoms. Although transcatheter thrombectomy in acute-phase DVT can reduce PTS [2], the effectiveness of endovascular treatment (EVT) for chronic-phase DVT has not been established. Moreover, some patients exhibit acquired arteriovenous fistulas (AVF) in addition to chronic DVT. The most common causes of AVF are trauma, iatrogenic injury, neoplasms, and arterial aneurysm erosion [3, 4], with several reports also implicating DVT in rare instances [5, 6]. Venous hypertension is another suspected cause of AVF after DVT; however, the exact pathogenesis of AVF accompanying DVT and their treatment remain uncertain [6].

AVF are often complex in morphology and difficult to repair surgically. This report demonstrates a possible treatment option for chronic DVT and AVF without an arterial-side approach via a stent graft on the venous side instead of the conventional arterial and venous same-side approach.

## Case presentation

**Fig. 1:**
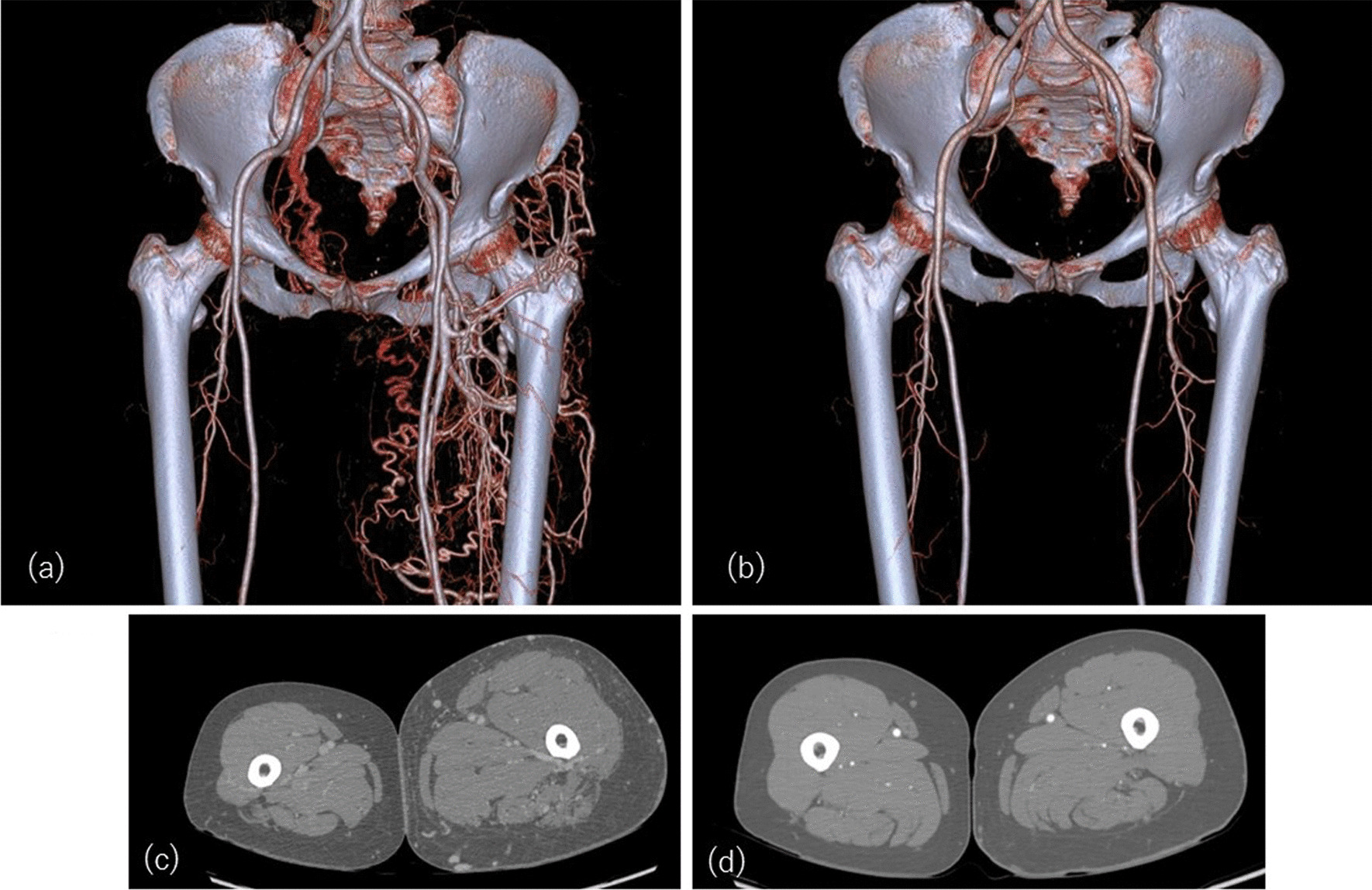
Development of collaterals and arteriovenous fistulas were evident in the left-to-femoral hypogastrium in preprocedure computed tomography angiography (**a**). Enhanced computed tomography showed significantly reduced collateral veins and arteriovenous fistulas 4 months after the procedure (**b**). Preprocedure enhanced computed tomography revealed marked arteriovenous fistulas and swelling (**c**). Postprocedure enhanced computed tomography showed reduced arteriovenous fistulas (**d**)

**Fig. 2:**
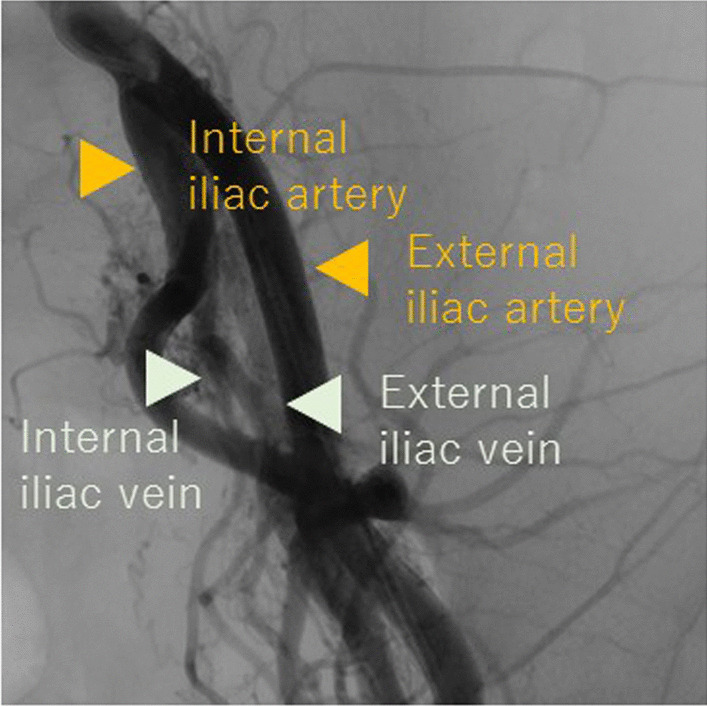
Preprocedure angiography showed the arteriovenous fistulas to run from the common and internal iliac arteries to the external iliac and common femoral veins

**Fig. 3:**
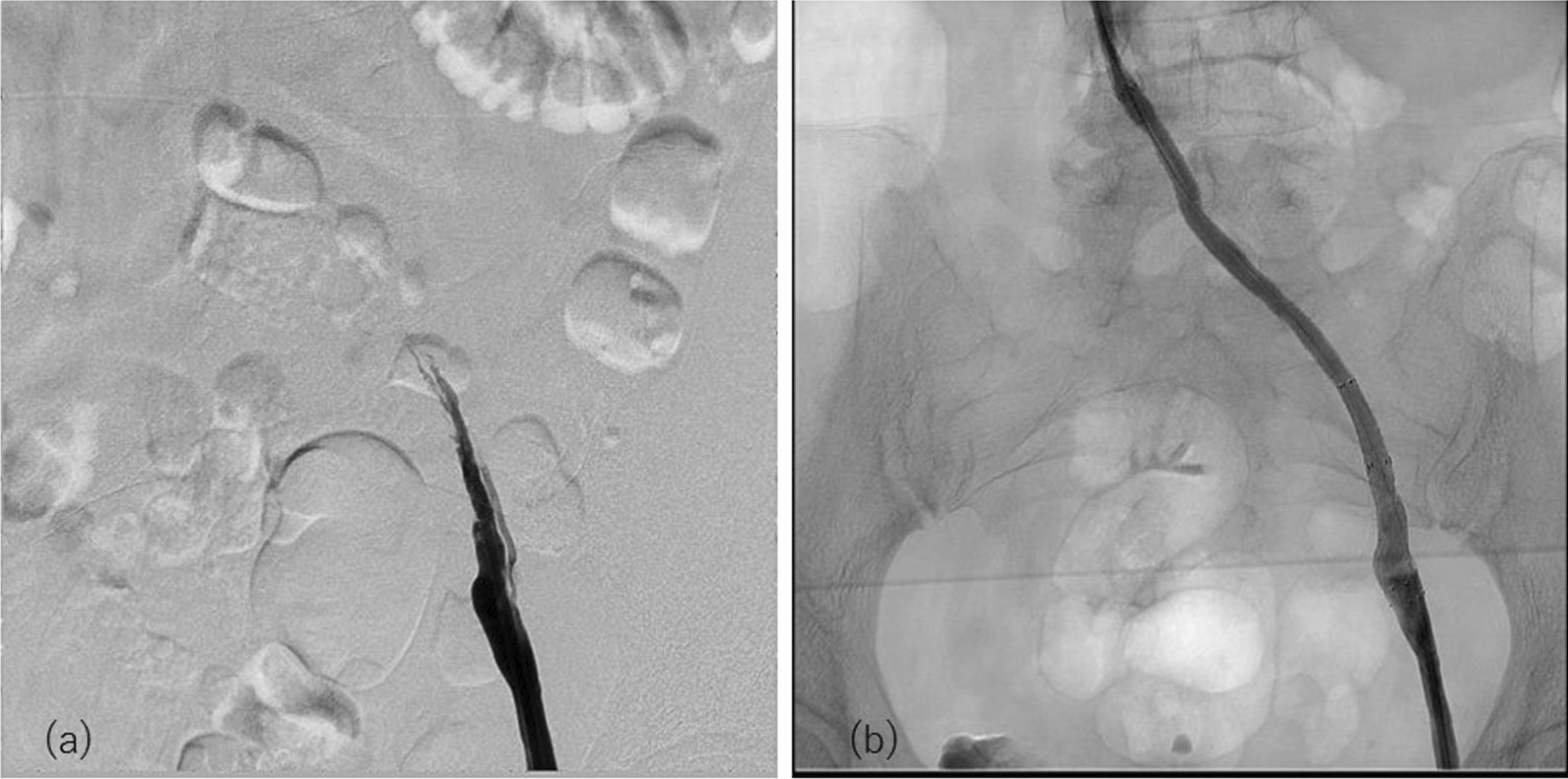
Preprocedural angiography of the left common iliac vein showed occlusion (**a**). Postprocedure angiography after deploying peripheral artery stents in the iliac vein. Good blood flow from the femoral vein to the inferior vena cava was achieved (**b**)

A 72-year-old Asian woman complained of recent severe left leg swelling and intermittent claudication at 200 m that had begun 2 years prior. Rivaroxaban was earlier started for an initial diagnosis of chronic DVT by contrast-enhanced CT. However, her symptoms did not improve after more than 1 year, so she was referred to our department for further examination. The patient had a medication history for depression, was gravida 2 para 2, and had received total hysterectomy for endometriosis. She had no smoking habit and no alcohol intake, no remarkable family medical history, and no exceptional socioeconomic background. Her medication included rivaroxaban 10 mg/day, spironolactone 25 mg/day, lorazepam 0.5 mg/day, eszopiclone 1 mg/day, duloxetine 40 mg/day, bromazepam 3 mg/day, pantothenic acid 300 mg/day, brotizolam 0.25 mg/day, olanzapine 2.5 mg/day, triazolam 0.25 mg/day, and magnesium oxide 750 mg/day. On physical examination, her heart rate was 72 bpm, blood pressure was 130/74 mmHg, body temperature was 36.2 °C, and SpO_2_ was 98%. Inspection of the left lower limb revealed swelling and edema with moderate tenderness and mild pain. The right lower limb appeared normal. There were no other findings on physical and neurological examination. Blood analysis showed TP 7.7 g/dL, albumin 4.8 g/dL, BUN 24.9 mg/dL, Cre 0.79 mg/dL, uric acid 6.3 mg/dL, total cholesterol 240 mg/dL, LDL cholesterol 125 mg/dL, HDL cholesterol 69 mg/dL, triglyceride 125 mg/dL, AST 27 U/L, ALT 18 U/L, γ-GTP 33 U/L, T-bil 0.9 mg/dL, ALP 114 U/L, LDH 169 U/L, CK 114 U/L, Amy 168 U/L, Na 141 mmol/L, K 4.5 mmol/L, Cl 104 mmol/L, CRP 0.10 mg/dL, Fe 101 µg/dL, HbA1c 6.0%, eGFR 45 mL/minute/1.73 m^2^, WBC 4.79 × 10^3^/μL, Hb 11.8 g/dL, Plt 29.7 × 10^4^/μL, PT 11.4 seconds, APTT 25.7 seconds, PT-INR 0.99, D-dimer 1.6 µg/mL, HCV antibody 0.1 C.O.I., HIV Ag/ab 0.1 C.O.I., HBs antigen 0.001 IU/mL, anti-cardiolipin IgG antibody < 8 U/mL, lupus anticoagulant 1.09, protein S activity 104%, and protein C activity 149%. Urine findings were pH 6.0, SG 1.004, PRO (−), LEU 1-4/HPF, BLD (−), NIT (−), KET (−), and URO < 2.0 mg/dL. Enhanced computed tomography (e-CT) revealed left common iliac vein occlusion, vein-to-vein collateral formation, and multiple AVF from the common iliac and internal iliac arteries to the external iliac and common femoral veins (Fig. 1a, c). Her symptoms worsened despite the commencement of direct oral anticoagulants (DOAC) and standard compression therapy at her previous hospital. Angiography confirmed the AVF to run from the common and internal iliac arteries to the external iliac vein (Fig. 2), with left external iliac vein flow proximally to distally. Ultrasound findings disclosed high-velocity arterialized waveforms in the external iliac and femoral veins. We first considered surgical repair for the AVF but opted against it due to the number of fistulas and their very complicated morphology. Her symptoms became progressively worse, which necessitated EVT from the common femoral veins for the occluded left iliac vein after repeated discussions among the vascular surgeon, plastic surgeon, and cardiologist. The patient provided sufficient informed consent for treatment before and after the procedure. We administered heparin 5000 units at the start of the procedure and maintained ACT at approximately 300 seconds. The procedure required a high tip load guidewire, such as Halberd^®^ (ASAHI INTEC, Aichi, Japan) or Chevalier tapered 30^®^ (NIPRO, Osaka, Japan), due to the very hard occlusion in the lesion. We completed successful wiring from the common femoral vein to the vena cava. Proximal protection to avoid a thromboembolism was deemed unnecessary in this case. After passing the wire, we were unable to get confirmation of the wire resting in a vessel by intravascular ultrasound. The patient had no pain, and her vital signs were stable. However, we decided to use a stent graft on this lesion for safety and to maintain blood flow. After balloon dilatation, we inserted two stents (VIABAHN^®^ [W.L.GORE & Associates, Inc. AZ, USA] 6.0 × 100 mm and SMART control^®^ [CARDINAL HEALTH, OH, USA] 10 × 40 mm) with slight overlap. Direct catheter thrombolysis was not needed since the final venogram showed good venous blood flow from the distal vein to the vena cava (Fig. 3b). Rivaroxaban 10 mg and aspirin 100 mg were continued after the procedure. She reported an improvement in fatigue in the left lower extremity the day after surgery, and her left leg swelling and intermittent claudication gradually ameliorated. Four months later, e-CT showed significantly reduced vein collaterals and AVF (Fig. 1b, d). At 6 months postoperatively, her laterality of femoral diameters and symptoms had almost disappeared. We confirmed patency of the left iliac vein 1 year after the procedure.

## Discussion

Switching from acute to chronic DVT is not uncommon. In some cases, symptoms improve after the development of a fistula, although swelling or tiredness of the lower limbs may remain. In the present case of AVF in DVT, revascularization was successful for reducing venous collaterals and AVF and for improving symptoms. DVT is considered a rare cause of AVF [7]. The exact pathogenesis of such acquired AVF remains unclear, although venous hypertension is regarded as a primary source [8]. The unique aspect of this case was that stent graft placement was performed on the venous side for repair of DVT with AVF. Although the usage of the stent was off-label, the procedure successfully improved the patient’s symptoms. Careful monitoring is needed regarding the long-term efficacy of this treatment.

We first suspected that revascularization was needed for both the occluded common iliac vein and the AVF in the present case. However, surgery for the AVF was considered difficult due to their complicated morphology. Since her symptoms were progressive, we opted for revascularization of the left iliac vein. At that time, we predicted that successful revascularization would improve her symptoms. A heavy tip load guidewire was required, and we could not exclude the possibility of extravessel wiring. Ultimately, we used a stent graft strategy for the chronic total occlusion lesion, resulting in vessel sealing on the venous side.

Venous hypertension-relieving therapy, including iliac vein revascularization and femoro-femoral bypass and embolization, may be useful for reducing AVF [8, 9]. The size reduction of AVF may produce shrinking and even closure of the arteriovenous shunt from the decrease in venous pressure [8, 10]. In this case, revascularization with a stent graft on the venous side was an effective treatment for AVF in DVT and might be a therapeutic option in patients unresponsive to standard compression therapy and anticoagulants.

There remain several clinical questions after this procedure, such as on the appropriate antithrombotic management of a stent graft. As some thrombosis in the left femoral vein remained, we are continuing DOAC and low-dose aspirin. Her symptoms have dramatically improved, with slight residual laterality. There is also some uncertainty on the outcome differences of a stent graft, bare Nitinol stent, or balloon dilatation with direct thrombolysis for chronic-phase total occlusion of iliac vein.

The precise effects of EVT for chronic venous thrombosis have not yet been established. As stents cannot be used for veins with EVT in Japan, revascularization was performed to improve the patient’s venous hypertension, symptoms, and AVF. The literature on vein disease treatment is currently limited. Therefore, additional cases of symptom resolution via EVT such as the current one are needed.

## Conclusion

DVT with AVF is rare, with few therapeutic options for chronic-phase DVT. In the present case, EVT of a common iliac occluded lesion with a stent graft was very effective in improving symptoms. EVT may therefore be a suitable option from the venous side for intractable chronic DVT with AVF.

## Data Availability

Data are available from the authors upon reasonable request.
